# The Role of Radioiodine Therapy in Differentiated Thyroid Cancer Arising from Struma Ovarii: A Systematic Review

**DOI:** 10.3390/jcm13247729

**Published:** 2024-12-18

**Authors:** Pietro Bellini, Francesco Dondi, Valentina Zilioli, Elisa Gatta, Maria Cavadini, Carlo Cappelli, Gian Luca Viganò, Francesco Bertagna

**Affiliations:** 1 Nuclear Medicine, ASST Spedali Civili di Brescia, 25123 Brescia, Italy; bpietro.bellini@asst-spedalicivili.it (P.B.); valentina.zilioli@asst-spedalicivili.it (V.Z.); 2 Nuclear Medicine, ASST Spedali Civili di Brescia and Università Degli Studi di Brescia, 25123 Brescia, Italy; francesco.bertagna@unibs.it; 3 Unit of Endocrinology and Metabolism, ASST Spedali Civili di Brescia and Università Degli Studi di Brescia, 25123 Brescia, Italy; elisa.gatta@unibs.it (E.G.); m.cavadini@unibs.it (M.C.); carlo.cappelli@unibs.it (C.C.); 4 Clinical Engineering, ASST Spedali Civili di Brescia, 25123 Brescia, Italy; gianluca.vigano@asst-spedalicivili.it

**Keywords:** struma ovarii, differentiated thyroid cancer, systematic review, radioiodine

## Abstract

**Background**: Struma ovarii (SO) is an ovarian teratoma with the presence of ectopic thyroid tissue. Differentiated thyroid cancer (DTC) in SO is a rare finding. Management of DTC in SO is currently not clearly established. We performed a systematic review of the literature to assess the role of 131I radiometabolic therapy in the treatment of DTC in SO. **Methods**: a wide literature search in the Scopus, PubMed/MEDLINE, and Web of Science databases was made to find published articles regarding the treatment of patients with DTC and SO. The quality assessment of studies was performed by QUADAS-2 evaluation. **Results**: eleven studies were included in the systematic review. All of them were retrospective studies and/or case series, and two of them also included a review of the literature. Most of the studies describe cases of DTC in SO treated by total thyroidectomy (TT) and subsequent radioiodine (RAI) therapy, especially in patients with distant metastases and/or concomitant thyroid cancer. However, the majority of patients apparently did not require radiometabolic therapy. **Conclusions**: TT and subsequent RAI therapy is usually performed in metastatic disease, not recommended in patients with intraovarian disease without risk factors, and it appeared possible but not mandatory in patients with risk factors.

## 1. Introduction

Ovarian teratoma (OT) is a germinal cell tumor of the ovary that contains different ectopic tissues, and the presence of thyroid tissue is frequent. Struma ovarii (SO) was first described by Boettlin in 1889 and reported by Gottshalk [1] and is defined as an OT with the presence of thyroid tissue involving more than 50% of the lesion. It represents a rare finding regarding less than 5% of the OT [2]. Malignant SO (MSO) is also rare and could be represented by differentiated thyroid cancer (DTC), highly differentiated follicular carcinoma (HDFC) that is a specific type of follicular neoplasia with histological characteristics of a benign lesion but aggressive behavior involving tissue outside the ovary, carcinoid and other thyroid cancer type [3,4]. DTC is the most frequent finding in MSO, particularly papillary thyroid cancer, representing about 44.8–62.5% of them [3,4,5], and it is, in most cases, confined to the ovary. Although DTC in MSO appears as a tumor with a good prognosis, and recrudescence after surgical resection is rare [5,6], several patients could present metastases at the diagnosis or in the follow-up (FU) [7,8] and, also due to its rarity and possible involvement of multiple professional figures, currently there are no shared guidelines focusing on the management of the DTC in MSO.

Nevertheless, DTC developed in the thyroid gland has well-established management described by several international guidelines that are represented by initial treatment with surgery followed in specific cases by radioiodine therapy (RAI) [9,10,11]. Particularly, the American Thyroid Association guidelines [9] suggest the use of RAI in cases of DTC with metastases, with a high risk of disease recrudescence, and the possibility to consider the treatment also in patients with intermediate and low risk of disease relapse. In addition, FU and further treatment are guided by the persistence of biochemical and/or structural disease [12,13,14], the 131I avidity of the malignant tissue [15,16,17,18], and the results of other diagnostic imaging examinations, such as ^18^F-fluorodesoxyglucose ([^18^F]FDG) positron emission tomography/computed tomography (PET/CT) [19,20].

The aim of this systematic review (SR) is, therefore, to evaluate the role of RAI in the treatment strategy of patients with DTC in SO, focusing in particular on describing the target of patients that could benefit from this therapeutic approach.

## 2. Materials and Methods

This systematic review was performed according to the “Preferred Reporting Items for a Systematic Review and Meta-Analysis” (PRISMA 2020 statement), employed as a guide in its development [21]. The complete PRISMA checklist is in the Supplementary Material. Pre-registering was not carried out.

### 2.1. Search Strategy

A comprehensive literature search in the Scopus, PubMed/MEDLINE, and Web of Science databases was performed to identify published articles on patients with DTC growth in SO. The algorithm used to search the articles was (“struma”) AND (“ovarii”) AND (“thyroid”) AND (“cancer”): due to the rarity of the disease, we chose to search all articles focusing on DTC development from SO, subsequently extracting only papers that focus on the possible role of RAI therapy. The search was updated to 30 June 2024, and no date limit was considered for the beginning. Articles in languages other than English were not included in the review. Despite the rarity of the disease, considering the systematic nature of the review, only original articles and case series with at least four patients affected by the disease were included. Instead, conference relations, case reports, reviews, preclinical studies, or editorials were not considered. Hybrid articles (e.g., case series plus review and/or retrospective cohort studies plus review) were only considered if at least four patients were included in the original part; these criteria were used to ensure the experience of the authors in the specific field in the selection, distribution, and outcome of the patients. In addition, the references of the retrieved articles were screened to find relevant articles to include in the review.

### 2.2. Study Selection

The titles and abstract of the selected articles were independently reviewed by two researchers (P.B., F.D.) who subsequently read the full-text version of the articles in the field of the review. Articles without unanimous consent were evaluated by a third expert physician (F.B.).

### 2.3. Quality Assessment

The quality assessment of the studies selected was performed using the Quality Assessment of Diagnostic Accuracy Studies version 2 (QUADAS-2) evaluation [22]. Quality assessment was performed independently by two reviewers.

### 2.4. Data Extraction

Each study included in the review was carefully evaluated, and the basic data extracted (author names, design of the study, country of origin, years of publication, number of patients, etc.…). The results section reported the main findings of every article considered in the review.

### 2.5. Literature Search Findings

**Figure 1:**
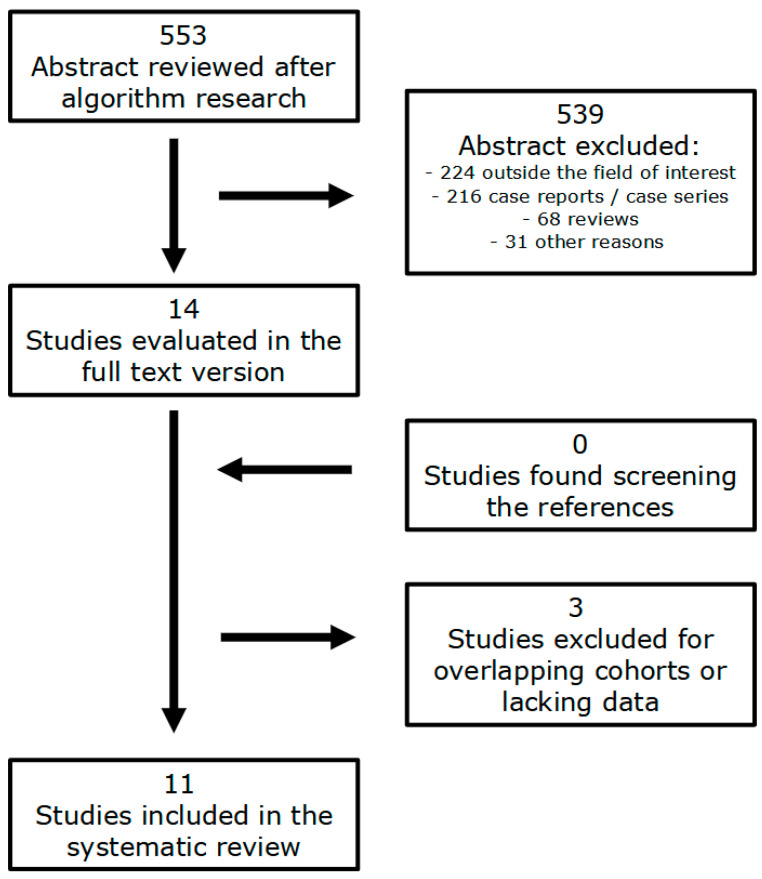
Flowchart of the research of eligible studies about patients with DTC in SO.

The literature search identified a total of 553 articles. After evaluating the titles and the abstracts of each study, 224 of them were excluded because they were not in the field of interest of the review, 216 because they were case reports and/or case series with less than 4 patients, 68 because they were reviews and 31 for other different reasons (language different than English, other type of documents such as Editorials, communications, book chapters and article corrections, hybrid studies where the selection and/or distribution of patients is not clear etc.…). Fourteen articles were consequently selected, and the full-text version of each one was retrieved [3,5,6,7,23,24,25,26,27,28,29,30,31,32]. Li S et al. wrote 3 of the 14 papers included in the review: the articles of Li S et al. considered the same group of patients, and consequently, we selected only the most recent paper [5,28]. Furthermore, the study of SC Yoo et al. presented some important data lacking and/or incongruence (e.g., the reporting of different numbers of patients in the sample between the abstract and the main document, lack of specification of the type of malignancy in MSO cases, etc.…) and could not be considered for the SR [32]. Finally, we included in the systematic review 11 articles [3,6,7,23,24,25,26,27,29,30,31]. After screening the references of the selected studies, no other articles were found to be suitable for inclusion in the review (Figure 1).

## 3. Results

**Figure 2:**
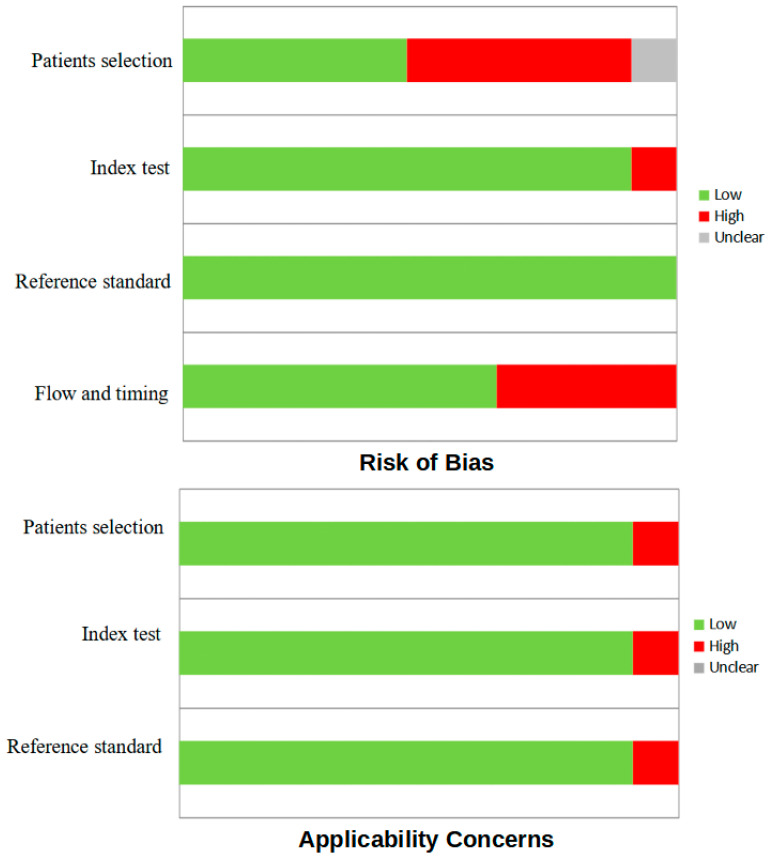
QUADAS-2 quality assessment for risk of bias and applicability regarding the studies included in the systematic review.

Quality assessment using QUADAS-2 showed that some studies had a high risk of bias in the applicability of patient selection due to the numerical limitation of the samples considered [24,26,29,30,31]. In addition, all studies had limitations in sequence and timing due to the retrospective design and variability in patients’ follow-up, which were not considered relevant. Moreover, with regard to flow and timing, 13.33% of patients in the study by Ryu HJ et al. were lost at the follow-up [23]. Finally, the study by Goffredo P et al. [6], considering the aim of the systematic review, presented a high risk of bias in the applicability of all items due to the lack of some information (e.g., histological characteristics of MSO and FU results), but remained useful due to its large sample and a clear indication of the number of RAI treatments performed. The result of the quality assessment is shown in Figure 2.

All the 11 studies included in the SR had a retrospective design [3,6,7,20,21,22,23,24,26,27,28], and 2 of them were integrated by a review of the literature [26,27]. Table 1, Table 2, Table 3 and Table 4 describe the main characteristics of the studies included in the review, and their main results were also reported.

Wei S et al. [3] described a single-center experience in the diagnosis and management of SO, specifically 11 patients with DTC growth in SO. PTC was the predominant histotype (90.6%), and only one patient was diagnosed with highly differentiated follicular cancer of ovarian origin (HDFCO). Only one of the PTC patients and the patient with HDFCO had extraovarian local spread at diagnosis, with the first one developing liver metastases during the FU and undergoing TT and RAI. Another one of the ten DTC patients underwent TT and RAI; the criteria for therapeutic choice were not explained. FU was adequate (mean 79 months) and negative in all these ten patients. Goffredo P et al. [6] described a large sample of patients obtained by consulting the Surveillance, Epidemiology, and End Results (SEER) database, in particular a selection of 68 patients with MSO defined as “histologically identical to differentiated thyroid carcinoma” considering the World Health Organization (WHO IV) edition of 2003. Unfortunately, the histology was not specified; however, the study described 6 cases of RAI treatment (9.2%), five with RAI alone and one with RAI plus external beam radiation therapy (EBRT). Again, three patients who received RAI had concomitant and/or secondary thyroid cancer. The duration of FU was variable (mean = 96 months, ranging from 2 to 408, standard deviation = 87.6), and the prognosis was excellent (overall survival ratio > 94% at 10 years). Garg K et al. [7] described a sample of 10 patients based on a single-center experience. In particular, eight patients presented with DTC and two patients with poorly differentiated cancer (PDC). Two patients who developed metastases during follow-up underwent TT and subsequent RAI: the first one had a right ovarian mass and cul-de-sac and omentum involvement at diagnosis but underwent RAI shortly after left adnexal involvement and second major abdominal surgery; the second one underwent RAI after disease recrudescence with peritoneal, liver and cul-de-sac involvement and because of high thyroglobulin levels. Both patients were initially diagnosed with benign SO and were still alive after six years of follow-up. Notably, three patients with classic risk factors in DTC, one with focal vascular invasion, one with surgical margin involvement, and one with infiltrative borders were treated with local surgery only. FU (mean 4.5 years) was negative in the eight patients without metastases. Ryu HJ et al. [23] presented a retrospective study based on a monocentric experience with 15 cases of MSO. Papillary DTC was the prevalent histotype (46.7%). Three patients presented with peritoneal metastases at diagnosis, and four patients had a history of or were subsequently diagnosed with thyroid cancer. Four patients underwent TT, and three of them received RAI, one for MSO metastases and two because of the presence of thyroid cancer. Notably, one patient with peritoneal metastases underwent only abdominal surgery because she was looking for pregnancy. Two patients were not available for follow-up (FU) assessment and the remaining 13 had a mean FU of 33 months. Of these 13 patients, 11 had negative results, 1 had stable disease, and one experienced disease progression with peritoneal seeding. The latter patients underwent both RAI. Gadducci et al. [24] presented a retrospective study based on a multicentre experience of 23 patients with malignant ovarian teratoma, in particular 5 patients with DTC in SO. Papillary DTC was the predominant histotype (60%). All patients had a stage I disease, according to the International Federation of Gynecology and Obstetrics (FIGO), and none of them had metastases at the subsequent follow-up. One patient had a previous history of thyroid cancer treated by TT and RAI, but none of the patients carried out RAI after the diagnosis of DTC developed from SO, and the FU (mean 60 months) was negative in all of them. Li S et al. [25] described a single-center experience in a retrospective study focusing on patients with ovarian strumal disease, specifically 13 patients with DTC in SO. Again, PTC was the most common histotype (46.2%). Three patients had metastases at diagnosis, and six subjects received adjuvant therapy: five received chemotherapy, while one received TT + RAI. The last patient had a partial response and was alive at the end of FU. In addition, three patients relapsed during FU: 1 died after surgical resection and chemotherapy, and two received TT + RAI and achieved a partial response and a complete response, respectively. Addley S. et al. [26] reported a retrospective monocentric experience analyzing 11 patients with MSO, and 6 of them presented DTC in SO. All of the patients had a PTC, and none of them presented metastases at diagnosis. Three patients with high risk, defined by aggressive histopathological features of PTC, a significant (>15 mm) disease deposit, close surgical margins, and/or lympho-vascular invasion, performed TT and further RAI. None of the subjects had disease relapse and/or died during the FU. The study of Devaney K et al. [27] described a retrospective analysis based on a monocentric experience of 54 cases of SO, particularly 13 cases of DTC in SO. Eleven patients had a PTC and 2 FTC in SO. There was only one case of peritoneal involvement without other metastases: the patient was treated conservatively only with abdominal surgery and had a partial response. No patients performed TT + RAI, and all of them did not develop disease relapse during the FU (average 87.6 months). Roth LM et al. [29] presented a case series of four patients from two different centers. Three patients had PTC, all treated with surgery, with one death apparently unrelated to the disease. The last patient had a poorly differentiated FTC invading the uterine wall and also involvement of the peritoneal tissue. This subject was unsuccessfully treated with gynaecological surgery, adjuvant chemotherapy, and TT + RAI and died three years after diagnosis. Marti JL et al. [30] presented the cases of 4 different patients treated for DTC arising from SO in a single-center experience. Three patients had PTC and one FTC, all of them had the intra-ovarian disease and underwent gynaecological surgery. One patient with 5 cm ovarian PTC also underwent TT + RAI, with the discovery of a 5 mm thyroid cancer with extra-thyroid extension and metastases in central lymph nodes; the criteria for therapeutic choice were not explained. During a median FU of nine years, no patients died and/or had disease recurrence. Poli R et al. [31] described a multicentre experience of 6 DTC arising from SO in six different patients, analyzing their genetic characteristics by next-generation sequencing. Five of them were intra-ovarian PTC, all <1 cm and without risk factors, and were treated with gynaecological surgery only. One patient presented with a 3 cm PDC with vascular invasion and was therefore treated with a prophylactic TT + RAI (3.7 Gbq) with a complete response. No patients had disease recrudescence and/or died during the FU (mean 104 months).

### Study Synthesis: RAI Applications and Outcome

A total of 155 cases of DTC in SO were collected in the eleven studies included in the present systematic review. TT + RAI was performed in 22 patients (14.19%): in particular, in seven cases, it was proposed to patients with risk factors (e.g., vascular invasion, extraovarian local spread, dimension), in two cases in patients with concomitant thyroid cancer and in seven cases in patients with metastatic disease. The clinical reason for TT + RAI therapy in the last six patients described in the study by Goffredo P et al. [6], such as their specific outcome, were not reported. Considering the other 16 patients treated with RAI, only one patient died due to disease complication or evolution (6.25%), and it was a subject with metastatic PDC. The seven patients with risk factors and the two with thyroid cancer achieved no evidence of disease (NED) at the end of their follow-up, whereas the other six subjects with metastases had a partial response in five cases and a complete response in one case.

## 4. Discussion

DTC in MSO represents a rare occurrence and a significant clinical challenge in its treatment, in particular, because of the absence of clear and well-established guidelines for its management. Most single-center or professional experience consists of a limited number of cases, particularly with regard to the role of the nuclear medicine physician and the possibility of RAI treatment. In our review, only a small proportion of patients with a diagnosis of DTC in SO received TT + RAI (14.19%), and, in particular, at least one of the 155 subjects described did not receive therapy despite peritoneal metastases: other literature reviews, as we could read later, reported greater use of RAI therapy in MSO disease. The outcome of patients treated with RAI could be considered good, with only one death in the only subject with poorly differentiated disease: the poor response to RAI treatment and prognosis of poorly differentiated thyroid cancer is unfortunate, well-known data [33,34]. Regarding the literature in favor of RAI treatment, a recent review by Cui Y et al. [4] considered 144 cases of MSO described in the literature: they reported 32.14% of patients with DTC in SO treated with TT + RAI, a figure higher than in our review (14.19%) probably because they also included case reports and a possible reduction in mortality in patients treated with RAI (1.5% less than in patients who did not receive it), but without statistical significance. DeSimone CP et al. [35] also described a possible larger use of RAI in the management of DTC arising in SO, reporting 24 cases with eight recurrences during follow-up, all in the group of patients who did not receive this therapy. Furthermore, Irena Batog W et al. [36] proposed to consider RAI therapy in patients with suspected and/or diagnosed concomitant thyroid cancer. Instead, a review by Li S et al. [37] considered 194 cases of MSO reported in the literature, 65 (35.6%) of which were treated with TT + RAI, and reported no significant correlation between RAI therapy and OS. However, the author’s consideration of the poor prognosis of patients with PDC described in the study is noteworthy, with the hypothesis of a correlation with the known poor response to RAI therapy in this type of disease. Another consideration to underline is the possible use of RAI in patients with advanced-stage diseases that are intrinsically characterized by poor prognosis: in this setting, the lack of statistical difference between patients treated with this therapy and those not treated could be read as in favor of RAI therapy. On the same side, considering only patients with metastases, Robboy et al. [38] described 27 cases of women with metastatic MSO, partly reported in the literature partly seen by the authors. In particular, twelve subjects (44.44%) received I131 with the worst outcome: five patients (41.67%) had a disease-related death, but one received RAI terminally (one month before death) and after chemotherapy, and three had an OS > 25 years anyway. The remaining group of 15 patients who did not receive RAI was characterized by five disease-related deaths (33.33%), but it is noteworthy that the FIGO stage was I or II in 75% of this group of patients, with some metastases treatable with major abdomino-pelvic surgery, instead only 50% in patients who received RAI. Lastly, Egan C et al. [39] proposed a risk stratification model by adapting the 2015 American Thyroid Association risk guidelines for MSO patients, considering parameters such as the presence of metastases, extraovarian extension, lymphovascular invasion, lymph node status, surgical margins, tumor size, and grade, similarly as proposed by Addley S et al. [26]: they found that the stratification obtained showed a good correlation between high-risk class and OS, but a poor correspondence with use of RAI therapy, as usually performed in classical thyroid cancer. In fact, only 30% of patients classified as high-risk in the National Cancer Database (NCDB) and 41% of patients in the Surveillance, Epidemiology, and End Results Registry (SEER) were treated with RAI. The authors highlighted this discrepancy in their conclusion and suggested further studies to determine how patients could benefit from this therapy. In general, RAI was suggested in fewer patients (19 to 158, 12.03% of patients from NCDB and 18 to 95, 18.95% of patients from SEER). Despite some studies describing the possible correlation between BRAF mutation status and the presence of DTC in SO [4,40,41], no papers described and/or analyzed the possible relationship between BRAF mutations and patient prognosis and/or the possibility of performing TT + RAI. To summarize and look at the literature, TT + RAI was not routinely proposed, and the percentage of patients treated with RAI ranged from 12.03% to 35.6%. There is a general consensus, as occurred in classical thyroid cancer, to perform TT + RAI in patients with distant metastases, mandatory in patients without the possibility of radical major abdomino-pelvic surgery, also considering the absence of data on the possible use of target therapy in literature, actually limited by few cases reports [42,43]. The only exception could be represented by young patients with only intra-abdominal disease and looking for pregnancy, as described by Ryu HJ et al. [23]. The same therapeutic program was generally recommended for patients with concomitant suspected or confirmed thyroid cancer [4,36]. The possibility of proposing RAI in patients with potential risk factors for disease recurrence is still debated and not clearly established: the first problem is represented by the absence of clear, endorsed classifications of patients considering these risk factors; the second problem, stemming from the rarity of the disease, is the absence of a prospective study confronting patients treated with RAI and patients not treated. Considering the actual common management of classical thyroid cancer, it is presumable that at least a part of patients with risk factors could benefit from this therapeutic approach, as suggested by Addley S et al. [26], Smith LP et al. [44] and explored in the study by Egan C et al. [39]. In these patients, discussion in the multidisciplinary team (MDT) is probably indeed preferred, presenting both solutions (TT + RAI vs. conservative approach) and explaining the limited data on disease management. At the same time, a consensus between experts and the scientific community should be explored to establish a common line of treatment for this rare disease. Summing up, as we mentioned before, the possible use of RAI in DTC derived from SO is still debated since several issues are present when considering it, such as the absence of clinical guidelines and general professional experience in its management. In addition, different data reported in the literature suggest that this therapy could be considered in a considerable number of patients, particularly for patients with aggressive diseases such as the presence of metastases, in the case of relapsed neoplasms, or in the presence of aggressive pathological features. The limited prognostic data presented in this review seems to support these proposals. Our personal experience, even though limited, endorses these findings since only patients with risk factors have been treated with RAI and had a good prognosis during the follow-up. Lastly, it is useful to remark that an MDT approach is mandatory to achieve patient-centered treatment planning. Some limitations, due to the characteristics of the studies included, affect our systematic review. First, some of the studies are characterized by limited cohorts, a limitation intrinsically related to the rarity of the disease. In addition, some data essential for the identification of patients who could be treated with RAI appeared lacking in a few studies. Moreover, the different specializations of the physician responsible for the therapeutic management and planning of these patients could result in different therapeutic approaches. Lastly, all of the studies included in the review had a retrospective design. Based on these facts, no meta-analysis of the data retrieved could be performed.

## 5. Conclusions

The results of the systematic review suggest that RAI following TT in DTC derived from SO is typically considered for metastatic patients and in cases of disease relapse. Patients with low-aggressive histologies and intraovarian diseases required only a surgical approach. In the other cases, such as extraovarian extension of the disease, vascular invasion, and/or the presence of aggressive histology, RAI treatment appeared possible and should be discussed with the patient in an MDT setting.

## Figures and Tables

**Table 1:** General characteristics of studies included in the review, first part.

First Author	Ref. No.	Year	Country	N. Pts.	Age (Median) [Range]	DTC in SO:Other SO
Wei S	[3]	2015	USA	11	59 years [35, 72]	11:85
Goffredo P	[6]	2015	USA	68	43 years [16, 79]	68:0
Garg K	[7]	2009	USA	10	41.5 years [32, 64]	10:0
Ryu HJ	[23]	2024	South Korea	15	48 years [30, 70]	15:155
Gadducci	[24]	2019	Italy	5	na ^	5:0
Li S	[25]	2023	China	13	na ^	13:262
Addley S	[26]	2021	USA	6	na ^	na
Devaney K	[27]	1993	USA	13	50 years [30, 77]	13:41
Roth LM	[29]	2008	USA	4	51 years [26, 70]	4:0
Marti JL	[30]	2012	USA	4	44 years [43, 57]	4:0
Poli R	[31]	2020	Italy	6	64.5 years [52, 66]	6:0

^ Data provided for all of the patients included in the study. USA: United States of America; na: not available; N.: number; Pts.: patients; DTC: differentiated thyroid cancer; SO: struma ovarii.

**Table 2:** General characteristics of studies included in the review, second part.

First Author	Ref. No.	PTC:FTC of HDFC:Other	FU Time (Mean)	Pelvic/Abdominal Surgery:TT:TT + RAI	Intraovarian:Extraovarian at Diagnosis	M0:M1 at Diagnosis	NED After First Line Therapy
Wei S	[3]	10:1:0	79 months [1, 204]	9:0:2	9:2	10:1	11:0
Goffredo P	[6]	na	96 months [2, 408]	na:na:6	52:13 *	56:9 *	na
Garg K	[7]	8:0:2	54 months [12, 168]	10:0:0	10:0	10:0	8
Ryu HJ	[23]	7:4:4	33 months [4, 156] **	10:4:1 ***	12:3	12:3	11:2 **
Gadducci	[24]	3:2:0	60 months [38, 203]	4:0:1 ***	5:0	5:0	5:0
Li S	[25]	8:5:0	74.4 months [9.6, 348]	12:0:1	10:3	10:3	11:2
Addley S	[26]	6:0:0	na	3:0:3	6:0	6:0	6:0
Devaney K	[27]	11:2:0	87.6 months [24, 216]	13:0:0	12:1	13:0 ^^	13:0
Roth LM	[29]	3:1:0	na	3:01	3:1	na	na
Marti JL	[30]	3:1:0	108 months [9.6, 156]	3:0:1	4:0	4:0	4:0
Poli R	[31]	5:0:1	104 months [24, 240]	5:0:1	6:0	6:0	6:0

* Data on 65 patients; ** Data on 13 patients; *** 1 patient performed TT and RAI for DTC before the diagnosis of MSO; ^^ One patient presented peritoneal strumosis which was not considered metastatic disease. PTC: papillary thyroid cancer; FTC: follicular thyroid cancer; HDFC: highly differentiated follicular cancer; TT: total thyroidectomy; RAI: radioiodine; FU: follow up; M0: absence of distant metastases; M1: presence of distant metastases; NED: non evidence of disease; na: not available.

**Table 3:** General characteristics of studies included in the review, third part.

First Author	Ref. No.	Disease Relapse:No Disease Relapse During FU	RAI After Relapse
Wei S	[3]	1:10	na
Goffredo P	[6]	na	na
Garg K	[7]	2:8	2
Ryu HJ	[23]	0:11 ****	na
Gadducci	[24]	0:5	na
Li S	[25]	3:8 *****	2
Addley S	[26]	0:6	na
Devaney K	[27]	0:13	na
Roth LM	[29]	na	na
Marti JL	[30]	0:4	na
Poli R	[31]	0:6	na

**** Data on 11 patients, plus 2 patients were alive with disease; ***** Data on 11 patients NED after first-line treatment. l RAI: radioiodine; FU: follow up.

**Table 4:** Main results of studies included in the review.

First Author	Ref. No.	Setting	Main Findings	TT + RAI
Wei S	[3]	Describe the pathology of SO in a single center experience. 11 of 96 of them were characterized as DTC in SO	PTC was the most frequent variant of DTC in SO (90.9%). TT and further RAI were performed only in 2 patients. The only 1 PTC patients with metastases discovered during follow up was also the only 1 with extraovarian spread at diagnosis.	RAI was performed in 2 patients: one presented a PTC fullicular variant (FVPTC) with round ligament involvement at diagnosis, the other one a FVPTC of 2.5 cm at diagnosis
Goffredo P	[6]	Describe the pathology, treatment and prognosis of MSO in the large database of Surveillance, Epidemiology, and End Results (SEER). 68 Patient with MSO defined as histologically identical to DTC were selected.	Radiation was not commonly administered as part of the treatment algorithm: only 6 patient received RAI (5 alone, 1 with EBRT). Overall survival rates (OSR) at 5, 10, and 20 years were 96.7%, 94.3%, and 84.9% respectively.	RAI was performed in 6 patients, particularly in 3 patients with concomitant or subsequent thyroid cancer. Histology was not reported.
Garg K	[7]	Describe the histopathology of MSO in a single center experience. 10 Patients with DTC developed in SO were described.	TT and further RAI were performed in the only 2 patients with metastases and/or disease relapse. Three patients with risk factor were treated conservatively. No cases of concomitant thyroid cancer were detected.	RAI was performed in 2 patients with a FVPTC in SO who developed metastases. In one case whole body scan (WBS) after RAI reveal a large liver metastasis. Both were alive with disease at the end of follow up.
Ryu HJ	[23]	Describe the pathology of SO in a single center experience. 15 of 170 of them were characterized as DTC in SO	PTC variant was the most frequent variant of DTC in SO (46.7%). Four patients presented also thyroid cancer. TT was performed in 4 patients, 3 for the thyroid cancer and one for MSO with metastases. Further RAI was performed in 3 of these patients.	RAI was performed in 2 patients with thyroid cancer and only in one case of MSO with metastases. No patients died for MSO.
Gadducci	[24]	Describe the pathology of malignant mature cystic teratoma in a multicentre experience. 5 of 23 of them were characterized as DTC.	PTC was the prevalent histotype (60%), all patients had a stage I FIGO. One patient had a previous thyroid cancer and performed RAI for them. No patients performed RAI after diagnosis of DTC in SO.	No patients presented metastases and/or died for MSO. No RAI was performed
Li S	[25]	Analyzing the prevalence and pathology of ovarian strumal disease in a single center experience. 275 patients with ovarian stumal disease, particularly 13 with MSO, were selected.	PTC was the predominant histotype (46.2%), 3 patients had metastases at diagnosis. Six patients received adjuvant therapy, but only 1 patient received TT + RAI at diagnosis. Three patients had disease relapse and one of them who not performed RAI died.	RAI was performed in 1 patient with metastatic MSO at diagnosis and 2 with disease relapse: two of them had a partial response and one a complete response.
Addley S	[26]	Describe the pathology of MSO in a single center experience. 11 patients were analyzed, particularly 6 patients with DTC in SO.	Patient with high risk, defined by aggressive histopathological features of PTC, a significant (>15 mm) disease deposit, close surgical margins and/or lympho-vascular invasion could benefit by TT and further RAI. None of the patients had metastases at diagnosis and none relapsed and/or died during FU.	TT + RAI was performed in 3 patients classified as “high risk”.
Devaney K	[27]	Describe the pathology of MSO in a single center experience. 41 patients were analyzed, particularly 11 patients with DTC in SO.	PTC was the prevalent histotype (84.6%). All of the patients performed only surgical therapy. One patient presented peritoneal involvement, but no adjuvant therapy was performed.	No RAI was performed. No patients died for MSO.
Roth LM	[29]	Describe 4 cases of DTC in MSO occurred in two centers experience.	Three PTC and one poorly differentiated FTC in SO were reported. This last patient presented metastases at diagnosis and performed chemotherapy and RAI but died anyway after 3 years.	TT + RAI was performed in one patient with poorly differentiated FTC.
Marti JL	[30]	Describe 4 cases of DTC in MSO occurred in a single center experiences.	Three PTC and one FTC in SO were reported. One patient performed TT + RAI with the discovery of a pT3N1a thyroid cancer. All patient achieved NED after therapy, and none died.	TT + RAI was performed in one patient with complete response.
Poli R	[31]	Analyzed the genetic characteristics of 6 DTC arised in SO in a multicentric experience	PTC was the main histotype (80%). BRAF, JAK3 and NRAS mutations were found. All of the patients achieved NED after therapy and no disease recrudescence and/or death occurred during FU.	TT + RAI was performed in one patient with PDC in SO with vascular invasion with a complete response.

Abbreviations: DTC: differentiated thyroid cancer; SO: struma ovarii; MSO: malignant struma ovarii; PTC: papillay thyroid cancer; FVPTC: PTC follicular variant; FTC: follicular thyroid cancer; PDC: poorly differentiated carcinoma; TT: total thyroidectomy; RAI: radioiodine; WBS: whole body scan; FU: follow up; EBRT: external beam radiation therapy; NED: non evidence of disease.

## Data Availability

Data supporting the reported results can be found using the public PubMed/MEDLINE, Scopus, and Web of Science databases.

## Supplementary Materials

The following supporting information can be downloaded at: https://www.mdpi.com/article/10.3390/jcm13247729/s1, PRISMA Checklist.
